# Guidance for anti-VEGF intravitreal injections during the COVID-19 pandemic

**DOI:** 10.1007/s00417-020-04703-x

**Published:** 2020-04-23

**Authors:** Jean-François Korobelnik, Anat Loewenstein, Bora Eldem, Antonia M. Joussen, Adrian Koh, George N. Lambrou, Paolo Lanzetta, Xiaoxin Li, Monica Lövestam-Adrian, Rafael Navarro, Annabelle A. Okada, Ian Pearce, Francisco J. Rodríguez, David T. Wong, Lihteh Wu

**Affiliations:** 1 Service d’ophtalmologie, CHU Bordeaux, Bordeaux, France; 2 Inserm, Bordeaux Population Health Research Center, team LEHA, Université de Bordeaux, UMR 1219, F-33000 Bordeaux, France; 3 Division of Ophthalmology, Tel Aviv Medical Center, Sackler Faculty of Medicine, Tel Aviv University, Tel Aviv, Israel; 4 Department of Ophthalmology, Hacettepe University, Ankara, Turkey; 5 Charité – University Medicine Berlin, Berlin, Germany; 6 Eye and Retina Surgeons, Camden Medical Centre, Singapore, Singapore; 7 Global Medical Affairs Ophthalmology, Bayer, Basel, Switzerland; 8 Department of Medicine – Ophthalmology, University of Udine, Udine, Italy; 9 Department of Ophthalmology, Azienda Sanitaria Universitaria Friuli Centrale (ASUFC), Ospedale Santa Maria della Misericordia, Udine, Italy; 10 Istituto Europeo di Microchirurgia Oculare, IEMO, Udine, Italy; 11 Eye Center and Eye Institute, Peking University People’s Hospital, Beijing, China; 12 Department of Ophthalmology, Lund University Hospital, Lund, Sweden; 13 Instituto Microcirugia Ocular, Barcelona, Spain; 14 Department of Ophthalmology, Kyorin University School of Medicine, Tokyo, Japan; 15 Royal Liverpool University Hospital, Liverpool, UK; 16 Fundación Oftalmologia Nacional, Escuela de Medicina y Ciencias de la Salud, Universidad del Rosario, Bogotá, Colombia; 17 Unity Health Toronto – St. Michael’s Hospital, University of Toronto, Toronto, Ontario Canada; 18 Macula, Vitreous and Retina Associates of Costa Rica, San José, Costa Rica

**Keywords:** Retinal disease, Ophthalmology, COVID-19, Coronavirus, Recommendations, Vision Academy

## Abstract

**Purpose:**

There is an urgent need to address how to best provide ophthalmic care for patients with retinal disease receiving intravitreal injections with anti-vascular endothelial growth factor agents during the ongoing global COVID-19 pandemic. This article provides guidance for ophthalmologists on how to deliver the best possible care for patients while minimizing the risk of infection.

**Methods:**

The Vision Academy’s Steering Committee of international retinal disease experts convened to discuss key considerations for managing patients with retinal disease during the COVID-19 pandemic. After reviewing the existing literature on the issue, members put forward recommendations that were systematically refined and voted on to develop this guidance.

**Results:**

The considerations focus on the implementation of steps to minimize the exposure of patients and healthcare staff to COVID-19. These include the use of personal protective equipment, adherence to scrupulous hygiene and disinfection protocols, pre-screening to identify symptomatic patients, and reducing the number of people in waiting rooms. Other important measures include triaging of patients to identify those at the greatest risk of irreversible vision loss and prioritization of treatment visits over monitoring visits where possible. In order to limit patient exposure, ophthalmologists should refrain from using treatment regimens that require frequent monitoring.

**Conclusion:**

Management of patients with retinal disease receiving intravitreal injections during the COVID-19 pandemic will require adjustment to regular clinical practice to minimize the risk of exposure of patients and healthcare staff, and to prioritize those with the greatest medical need. The safety of patients and healthcare staff should be of paramount importance in all decision-making.

## Introduction

Intravitreal injection of anti-vascular endothelial growth factor (VEGF) agents is widely regarded as the standard of care for patients with retinal disease, including neovascular age-related macular degeneration and diabetic macular edema (DME) [1]. In these unprecedented circumstances, where the COVID-19 pandemic has overwhelmed many healthcare systems and there is a widespread implementation of strict measures to reduce its spread, there is an urgent need to support ophthalmologists who are treating patients receiving intravitreal injections, to help guide decision-making.

Risk factors for developing severe symptoms in response to infection with COVID-19 are common among patients with retinal disease and include being ≥ 65 years old, living in a nursing home or care facility, and having an underlying health condition, particularly if this condition is poorly controlled. Health conditions of particular risk include moderate-to-severe asthma or chronic lung disease, serious cardiovascular complications, being immunocompromised, diabetes, severe obesity, liver disease, and chronic kidney disease requiring dialysis [2].

Several organizations have now produced general guidance for ophthalmologists on managing patients during the pandemic, including the American Academy of Ophthalmology, the French Society of Ophthalmology, the German Ophthalmological Society, the Royal College of Ophthalmologists, the Japanese Ophthalmological Society, and others [3–12]. In particular, the Royal College of Ophthalmologists has developed highly granular guidance on the medical management of patients with retinal disease during the COVID-19 pandemic (Table 1) [7]. However, this guidance is specifically relevant to the UK healthcare system and its applicability outside of the UK is subject to local regulations, practice capacity, and other country-specific factors.

**Table 1:** The Royal College of Ophthalmologists’ medical retinal management plans during COVID-19 [7]

For patients already under review by the hospital eye service	For new patients
Wet AMD: Maintain all patients on 8 weekly anti-VEGF therapy with no clinic review unless they mention a significant drop in vision at their injection visit. Such patients may need OCT and visual acuity assessments and management changed, if deemed appropriate	Wet AMD: Diagnosis confirmed with OCT and OCT-A, if available. Confirmed new wet AMD cases should be treated with a loading phase of 3 injections of anti-VEGF and then continued on 8 weekly with no clinic review. Consent is taken on the day of first injection
DME: Defer anti-VEGF injections and review in clinic after 4 months. Exceptions are eyes with severe NPDR and active PDR that may require anti-VEGF agents and PRP. Virtual review with OCT and wide-field color photography is the preferred option to review these patients	DME: Defer treatment for 6 months unless associated with R3. R3 patients should be treated with PRP
BRVO: Defer review in clinic by 4 months	BRVO: Defer review in clinic by 4 months
CRVO: For patients with macular edema due to CRVO who have had at least 6 injections, consider PRP if required. Otherwise, review in clinic in 4 months	CRVO: Provide 6 mandated loading phases if visual impairment due to macular edema and then review in clinic. If, in the opinion of the clinician, there is no hope of visual improvement, an alternative approach is an extensive PRP laser to reduce the risk of rubeotic glaucoma. However, visual outcomes are likely to be poorer with this approach

Cited with permission from the Royal College of Ophthalmologists (RCOphth). The RCOphth COVID-19 team have prepared guidance as a temporary response to the clinical management of patients during the ongoing COVID-19 pandemic and in parallel with Public Health England COVID-19 guidance. These publications are not meant to substitute patient care under normal circumstances. RCOphth clinical guidelines are to be used for the standard long-term care of patients

These considerations are for the UK only and may not be valid for other countries

*AMD* age-related macular degeneration, *BRVO* branch retinal vein occlusion, *CRVO* central retinal vein occlusion, *DME* diabetic macular edema, *NPDR* non-proliferative diabetic retinopathy, *OCT* optical coherence tomography, *OCT-A* optical coherence tomography angiography, *PDR* proliferative diabetic retinopathy, *PRP* panretinal photocoagulation, *VEGF* vascular endothelial growth factor

Here, we discuss in more detail the key considerations for managing patients receiving intravitreal injections of anti-VEGF during this challenging time, taking into consideration the various national guidelines mentioned above. This article provides guidance on implementing steps to prioritize treatment for those with the greatest medical need and to improve safety to minimize the risk of infection in healthcare staff and patients.

## Methods

The Vision Academy is an international group of retinal physicians who work together to share existing skills and knowledge, and provide collective recommendations on clinical challenges in areas where there is a lack of conclusive evidence in the literature [13].

These recommendations were developed during a virtual meeting in March 2020 by the 14-member Vision Academy Steering Committee, who reviewed available guidance documents and debated the key challenges of managing patients receiving intravitreal injections of anti-VEGF agents during the COVID-19 pandemic. Following thorough analysis and discussion of relevant guidelines and documents, members submitted recommendations which were then collated and systematically refined before being voted on by the Steering Committee for consensus.

## General considerations: avoiding contamination and treating patients in a limited-resource environment

When considering these recommendations, recognize that as important as vision loss may be to patients, non-ophthalmic life-threatening situations must supersede ophthalmological considerations. The safety of patients and healthcare staff is of paramount importance in all decision-making.

### General guidance

As medical and healthcare personnel can be a source of contamination, they should be scrupulously monitored for signs of infection, swabbed, and, where required, quarantined according to national/institutional guidelines. Staff should be regularly retrained on safety practices, including proper use of personal protective equipment (PPE), to reduce the spread of COVID-19. Video guidance or regularly scheduled virtual meetings may be beneficial for this purpose. It is also essential that staff scrupulously follow personal, facility, and instrument hygiene/disinfection rules as per local guidelines.

Appointments of COVID-19-positive or suspected positive patients should be deferred until total resolution of symptoms or risk. However, ophthalmic emergency intervention or surgery due to imminent danger of blindness or severe vision loss in these patients should proceed in an appropriate setting with appropriate PPE. People with mild illness due to COVID-19 may present with typical symptoms of uncomplicated upper respiratory tract viral infection, such as cough, headache, fever, fatigue, muscle pain, anorexia, malaise, sore throat, dyspnea, or nasal congestion. Rare symptoms include nausea, vomiting, and diarrhea [14]. Non-urgent appointments should be postponed where there is capacity to reschedule within a reasonable time period. Consistency in the management and use of PPE throughout the patient journey is essential [15]; patients should wear a mask at all times to reduce the potential transmission of COVID-19 to healthcare staff or other patients [16].

The applicability of these considerations will depend on the current state of the pandemic in each individual country; however, the general principles should be relevant for all. It is vital that staff receive appropriate and regular training on COVID-19 transmission prevention and that safety measures to reduce the risk of transmission to both patients and healthcare staff are implemented. In countries where there are a large number of COVID-19 cases, postponement of non-urgent appointments may be appropriate to reduce the risk of exposure for patients and staff, particularly as patients are often elderly and may have conditions that make them vulnerable to more severe disease [2].

It is also essential during this time that there is sufficient communication with patients via letters or phone calls, in order to provide clear instruction. This communication should cover the status of their upcoming appointments, provide safety advice on how to minimize their risk of exposure, and advise on what they should do if they develop COVID-19 symptoms or notice deterioration of their vision. These steps should provide reassurance to patients and ensure that those requiring urgent treatment or monitoring for their vision attend their appointments. The Vision Academy has developed a template letter to patients that can be customized by ophthalmologists to explain the situation and reassure and guide patients throughout this crisis [17].

## Prioritizing patients according to medical need: assessing the risks and benefits

The strain on healthcare services imposed by the COVID-19 pandemic, together with government restrictions on movement and the need to limit any potential exposure to the virus, may necessitate the prioritization of appointments for those deemed to be at the greatest risk of vision loss should treatment be postponed.

### Guidance for prioritizing patients according to medical need

Diabetic and elderly patients are at a high risk for COVID-19 complications [2] and should not be exposed to avoidable risk; however, continuation of care where possible is important to avoid irreversible vision loss. In general, patients with neovascular age-related macular degeneration (especially those in the first 2 years of treatment), neovascular glaucoma, new cases with significant vision loss, new central retinal vein occlusion cases, and monocular or quasi-monocular patients (only one eye > 20/40) should be prioritized and their treatment schedules maintained.

Patients with DME and branch retinal vein occlusion are less likely to suffer irreversible vision loss in the short term [18, 19], and postponement of appointments for non-monocular patients may be considered (except for patients with significant vision loss from recent DME and patients in the acute phase of retinal vein occlusion). However, prolonged treatment postponement (> 4–6 months) should be avoided and the situation should be reassessed within 2–3 months depending on the stage of the pandemic and the confinement measures in place in each individual country*.* These considerations should be thoroughly discussed (remotely) with the patients and staff, taking into account the local legal/regulatory environment, status of the epidemic, and the capacity of each practice to reschedule postponed procedures. Indefinite postponement of appointments without rescheduling within a reasonable time period is inadvisable.

Prioritization of appointments may not be necessary in all centers or countries. These considerations are particularly appropriate for countries where there is a high transmission rate of COVID-19, in order to reduce the risk of exposure of patients and healthcare staff and to reserve appointments for the most urgent cases where resources are limited. Each case should be evaluated individually to balance the risk of exposure of often elderly and vulnerable patients to COVID-19 with the potential visual implications of treatment postponement. These risks should be discussed with the patient to ensure they are informed and comfortable with the approach.

## Considerations to reduce exposure during the patient visit

Reducing the exposure of patients and healthcare staff to COVID-19 is of paramount importance. Reports from several countries have indicated that significant numbers of healthcare staff have now contracted COVID-19 [20]. In addition, patients with retinal disease are often elderly and may have underlying health conditions that put them at increased risk of developing severe respiratory symptoms in response to COVID-19 [2, 21].

### Guidance to reduce exposure of healthcare staff and patients

Lowering the risk of exposure to COVID-19 can start before a patient visits the waiting room, by providing a letter detailing safety and hygiene measures or by contacting them on the phone prior to their appointment to screen for symptoms and advise them on next steps, as per local guidance. Hygiene measures should be enforced, and patients should be educated on the importance of physical distancing by 1 or 2 m [22, 23] and the potential benefits of wearing a mask [16]. This can be achieved by sending a “Dear Patient” letter to all patients [17]. If possible, pre-screen patients by phone to identify those who are symptomatic or suspected COVID-19-positive patients and direct them to an appropriate setting. This may include a designated section of the clinic or hospital with enhanced protection/disinfection measures and PPE as per local/regional guidelines.

Staff must wear PPE (including masks, gloves, goggles, and suits) for patients who are COVID-19-positive or suspected to be positive, or possibly for all patients, as directed by local authorities and institutions. An N95 or FFP2 mask is preferable wherever possible [24]; however, in many countries, availability may be limited or they may be reserved for use by first-line COVID-19 departments only. Where N95 or FFP2 masks are unavailable, a surgical mask should be worn by both the clinician and patient. In any case, the selection of appropriate PPE should be determined by local risk assessment and national authority guidance.

Limit exposure in waiting rooms by enforcing a 1- or 2-m distance [22, 23] between people, as per local guidance, and take steps to limit overall waiting room attendance. These steps could include spacing out appointments, permitting one accompanying adult only (and only if absolutely necessary), and allowing patients to queue outside the waiting room. Keep the examination as brief as possible, consider implementing distancing measures between patients and staff, and ensure good ventilation in all rooms to reduce any potential viral vector load. Other measures to reduce exposure during the appointment include limiting the use of special instruments and tests to only those critical for decision-making. It is also highly recommended to thoroughly disinfect hands and equipment between patients. Note that keyboards in examination rooms can be a source of contamination, so use scribes/remote keyboards/flat keyboards that are easily disinfected or cover keyboards with a disposable transparent film whenever possible.

Avoid thorough visual acuity testing of all patients. If needed, a simple test, ideally self-performed, may be sufficient (e.g., a near-reading chart). Consider performing brief visual acuity testing if an important vision change is reported, jumping to the smallest-achievable line rather than starting from the top. Limit optical coherence tomography examinations to cases where it may impact treatment decisions (i.e., it may not be required in stable patients under fixed regimens) and avoid using special instruments (e.g., tonometer/fundus camera/angiograph) unless they are absolutely critical to decision-making.

**Fig. 1:**
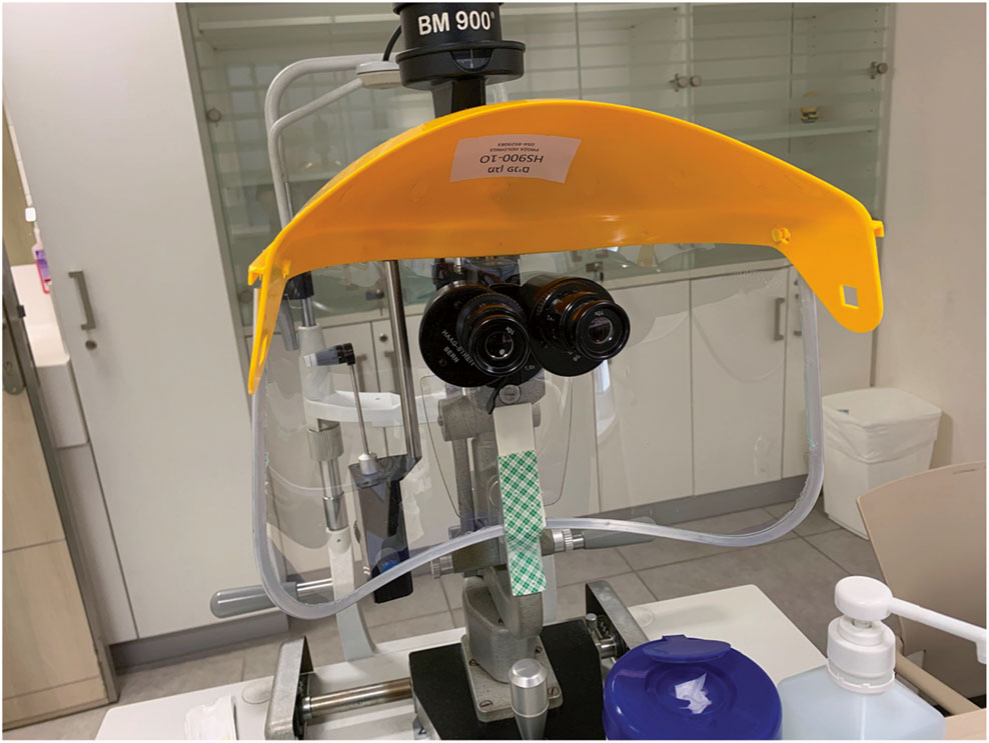
Example of a slit lamp set-up equipped with a protective shield (image courtesy of Professor Anat Loewenstein)

Fix large plastic/plexiglass shields on slit lamps, and, as there is increasing evidence that asymptomatic patients may be positive for COVID-19 (44% in Italy) [25] and up to 20% of physicians may also be positive, both patients and physicians should consider wearing a face mask during slit lamp examination (Fig. 1).

## Intravitreal treatment regimen considerations

Management of patients requiring intravitreal injections with anti-VEGF agents during the COVID-19 pandemic is a particular treatment challenge for ophthalmologists. Both the practicality and risk–benefit of a treatment approach should be carefully considered to minimize patient exposure and preserve sight.

### Guidance on anti-VEGF treatment regimens

While performing intravitreal injection procedures, safety measures should be implemented to limit exposure to COVID-19. These measures include use of masks by both the patient and physician, gloves, sterile drapes, and lid speculums, and keeping talking to a minimum where appropriate. To minimize exposure, the aim should be to preserve treatment visits rather than monitoring visits whenever possible.

In general, one should refrain from treatment regimens and regimen changes that require frequent monitoring to adjust dosing intervals:

- Do not switch treatment regimen unless there is a clear lack of response.
- Do not change treatment intervals in patients with neovascular age-related macular degeneration who are responding to a fixed-dosing regimen, if possible.
- For patients with age-related macular degeneration receiving variable-interval treatment regimens (treat-and-extend and PRN), consider reverting to the last effective treatment interval and use this for fixed dosing to minimize the need for monitoring.
- In new patients, maintain the loading phase schedule and select longer-acting therapies if possible.
- In patients with DME/retinal vein occlusion who are already on dexamethasone implants, consider reimplantation only if they are responding well and have a history of normal intraocular pressure under treatment.

Telemedicine consultations, where feasible, are beneficial and can help physicians assess which patients should attend the clinic in person. This could be particularly useful for monitoring patients who are at less risk of irreversible vision loss and can be deprioritized as described in the previous section. In such patients, monitoring on function only may be acceptable in the short term (not to exceed 4–6 months). In order to avoid in-person visits, equip and instruct such patients to self-monitor their vision (e.g., with Amsler grids or by reading texts with various font sizes). Where possible, implement and recommend the use of home monitoring technologies. The Vision Academy has previously reviewed a number of home monitoring technologies, including smartphone apps for vision monitoring [26]; however, it should be noted that these technologies may require patient training which may not be feasible at this time.

Under normal circumstances, treat-and-extend regimens are highly beneficial to reduce the number of intravitreal injections a patient requires. However, the additional monitoring appointments that these regimens often entail may make them unsuitable during this unprecedented time, where there is a need to limit patient exposure and free up resources. Similarly, treatment intervals and regimens should ideally be maintained for patients who are responding to them to limit the need for additional monitoring. Therefore, fixed dosing intervals with reduced monitoring should be considered.

## Treatment facility recommendations

Widespread implementation of measures to slow the rate of spread of COVID-19, including restrictions on movement for vulnerable groups or all residents, is now in force in several countries. This, together with the need to free up resources within hospital settings and identify appropriate ophthalmic treatment strategies for patients who are symptomatic, may require adjustment of the treatment facility for certain patients.

### Considerations for treatment facility organization

Consider implementing home care if and where feasible, particularly for patients under lockdown; home injections may be acceptable in some countries. Emergency surgery/intervention due to imminent danger of blindness or severe vision loss in symptomatic, confirmed, or COVID-19-suspected patients should proceed in an adequate facility with appropriate PPE, as per local guidelines. For asymptomatic/non-COVID-suspected patients who need treatment, referral to a non-hospital-based clinic or ambulatory surgical center may be preferable, especially in cases with high infection rates/medical facility shortage.

The feasibility of making changes to the treatment setting will vary widely from country to country. Where possible, offering home care to vulnerable patients or directing them to non-hospital-based clinics may reduce some of the pressure on overwhelmed hospitals. Offering these alternatives may also be reassuring for patients who may be fearful of attending a hospital at this time due to the potential exposure risks.

## Further considerations

It is essential to ensure that patients feel comfortable and reassured that their vision and well-being are being appropriately managed, despite possible treatment postponements and changes to the way in which their routine appointments are carried out.

### Considerations for reassuring patients

An emergency contact number manned by a senior ophthalmologist should be provided to offer consistent/appropriate patient-triaging advice. Also consider distributing advice and instructions for patients receiving intravitreal injections, for example through a letter addressed to all patients [17].

Fixed-dosing regimens reduce time spent in the clinic and have been shown in pivotal clinical trials to be an effective way of delivering treatment [19, 27, 28]. This should be reiterated to reassure patients who are used to a more individualized approach.

Many patients will be confused or uncertain about whether they should be attending their ophthalmology appointments during this time, and it is important to provide clear advice so that they feel supported and to ensure that those who require treatment receive it. Risk–benefits must be carefully weighed, discussed with the patient, and documented, while always considering the local legal and regulatory environment. Triaging of patients will likely be necessary in many centers, and any resulting postponement of appointments should be discussed with the patient, explaining the risks and benefits of the approach. However, take into consideration that reassuring patients that, in most cases (i.e., DME), vision will not be significantly adversely affected by interrupted/postponed treatment may have medico-legal issues. Offering a telephone number that they can call for advice should their vision deteriorate may also provide reassurance. More than ever, talk (remotely) to patients, explaining what is at stake, and make them an active partner in treatment decisions.

## Conclusion

Strategies for managing patients with retinal disease during this uncertain time should focus on minimizing the risk of exposure to COVID-19 for both the patient and healthcare staff and, where necessary, prioritizing treatment for those at the greatest risk of irreversible vision loss. In order to limit patient exposure, ophthalmologists should consider simplifying treatment regimens for patients receiving intravitreal injections and, where possible, refrain from using regimens that require frequent monitoring.

Adjustments to regular clinical practice will likely be necessary for many ophthalmic centers, and these should be continually reassessed as we progress through the current pandemic and as further guidance is provided by ophthalmological societies. In the meantime, by implementing stringent safety practices and triaging those who are most vulnerable, we have the opportunity to continue to provide the best possible care to patients.
